# CAR Therapies: Ex Vivo and In Vivo Potential of Exosomes and Biomimetic Nanoparticles

**DOI:** 10.3390/cancers17233766

**Published:** 2025-11-25

**Authors:** Ekaterina Tkachenko, Natalia Ponomareva, Konstantin Evmenov, Artyom Kachanov, Sergey Brezgin, Anastasiya Kostyusheva, Vladimir Chulanov, Elena Volchkova, Alexander Lukashev, Dmitry Kostyushev, Peter Timashev

**Affiliations:** 1 Laboratory of Genetic Technologies, Martsinovsky Institute of Medical Parasitology, Tropical and Vector-Borne Diseases, Sechenov University, Moscow 119435, Russia; ekaterinatkachenko03@mail.ru (E.T.); ponomareva.n.i13@yandex.ru (N.P.); kevmenov@mail.ru (K.E.); kachanov.av99@gmail.com (A.K.); seegez@mail.ru (S.B.); kostyusheva_ap@mail.ru (A.K.); 2 Engelhardt Institute of Molecular Biology, Russian Academy of Science, Moscow 119991, Russia; vladimir@chulanov.ru; 3 Laboratory of Experimental Therapy of Infectious Diseases, Martsinovsky Institute of Medical Parasitology, Tropical and Vector-Borne Diseases, Sechenov University, Moscow 119435, Russia; 4 Department of Infectious Diseases, Sechenov University, Moscow 119435, Russia; antononina@rambler.ru; 5 Martsinovsky Institute of Medical Parasitology, Tropical and Vector-Borne Diseases, Sechenov University, Moscow 119991, Russia; alexander_lukashev@hotmail.com; 6 Research Institute for Systems Biology and Medicine, Moscow 117246, Russia; 7 Faculty of Bioengineering and Bioinformatics, Lomonosov Moscow State University, Moscow 119192, Russia; 8 Institute for Regenerative Medicine, I. M. Sechenov First Moscow State Medical University, Moscow 119991, Russia; timashev_p_s@staff.sechenov.ru

**Keywords:** CAR-T therapy, T cells, biological nanoparticles, targeted delivery, gene therapy

## Abstract

Chimeric antigen receptor (CAR) therapy represents a promising modality for treating cancer and autoimmune diseases, employing genetically engineered immune cells. Despite remarkable clinical outcomes, its broad implementation is constrained by significant challenges, including toxicity, limited specificity, and complexities associated with genetic material delivery. Biological nanoparticles, such as exosomes, virus-like particles, and biomimetic nanostructures, possess unique properties that can address these limitations. These nanoplatforms enable targeted delivery of genetic constructs, mitigate the risk of cytokine release syndrome, modulate CAR cell activity, and can function as biosensors. Furthermore, they facilitate non-viral, in vivo CAR cell engineering, streamlining the process compared to conventional ex vivo methods. The advancement of in vivo strategies underscores the critical need to overcome toxicity hurdles inherent to current CAR-T platforms. In this context, exosomes and biomimetic nanoparticles offer considerable potential due to their innate biocompatibility, programmability, and versatile cargo capacity for payloads like mRNA and circular RNA. This review comprehensively outlines contemporary genetic platforms for CAR expression and examines the opportunities presented by biological delivery vehicles. It focuses on recent achievements and revisits fundamental CAR principles through the lens of emerging technologies aimed at confronting persistent challenges in the field.

## Simple Summary

The introduction of chimeric antigen receptors (CAR) into T-cells has proven highly effective against hematologic malignancies, frequently leading to complete remissions. In contrast, the success of CAR-T therapy in solid tumors has been modest. This review focuses on the emerging promise of biomimetic nanoparticles to overcome this hurdle by enabling highly efficient CAR-T cell programming, both ex vivo and in vivo. We synthesize the current landscape, reviewing the technological challenges, limitations, and ongoing clinical progress of this innovative strategy.

## 1. Introduction

Chimeric Antigen Receptor T-cell (CAR-T) therapy is an innovative form of immunotherapy that involves genetically engineering a patient’s own T-lymphocytes to express a surface chimeric antigen receptor (CAR). The structure of this synthetic receptor is the primary determinant of the therapy’s specificity, function, and overall efficacy [1].

The clinical success of CAR-T therapy has been most prominently demonstrated in treating relapsed and refractory hematological malignancies. Multicenter studies have reported unprecedented response rates, with complete remission achieved in 40–70% of patients across various lymphoma types [1]. Similarly, in patients with relapsed B-cell acute lymphoblastic leukemia, complete response rates of 71–81% signify a breakthrough for a population with limited treatment options [1]. The transition of CAR-T from clinical trials to mainstream medicine is confirmed by regulatory approvals, such as the FDA’s authorization of tisa-genlecleucel and axicabtagene ciloleucel, underscoring its translational significance [2].

A key advantage of CAR-T therapy is its capacity to induce a potent immune response even against cancers resistant to conventional treatments like chemotherapy and radiotherapy [3]. However, despite its remarkable efficacy in B-cell malignancies, the application of CAR-T therapy to solid tumors and non-oncological diseases faces substantial challenges [4,5].

The clinical application of CAR-T therapy is constrained by two major challenges: significant toxicity and limited long-term efficacy. Treatment-related adverse events are frequent and severe, with cytokine release syndrome (CRS) occurring in 77–89% of patients (24–46% experiencing grade 3 or higher) and immune effector cell-associated neurotoxicity syndrome (ICANS) affecting 30–60% of patients, with severe manifestations in up to 10–25% of cases [2].

The long-term effectiveness is also modest, with a 12-month progression-free survival rate of ~50% [2]. This limited efficacy stems from several factors, including poor tumor infiltration, the immunosuppressive tumor microenvironment, and target antigen heterogeneity. Furthermore, the mechanisms driving toxicity are intrinsically linked to the potent activation of CAR-T cells. The uncontrolled, constitutive signaling of chimeric antigen receptors (CARs) poses significant clinical risks. A primary concern is severe on-target, off-tumor toxicity, where sustained activity destroys healthy tissues expressing low antigen levels, as seen in CD19-mediated B-cell aplasia [6].

Persistent antigen exposure and tonic signaling drive T-cell exhaustion, characterized by the upregulation of inhibitory receptors (e.g., PD-1, LAG-3), impaired effector function, and reduced proliferative capacity. This exhausted state directly compromises long-term antitumor efficacy and contributes to relapse, establishing a direct link between the mechanisms of toxicity and treatment failure [7].

The potentially life-threatening toxicities of CRS and ICANS are of particular concern [8]. CRS is driven by uncontrolled CAR-T cell and macrophage activation upon target engagement, triggering a massive release of pro-inflammatory cytokines (e.g., IL-6, IFN-γ, GM-CSF). This creates a cascade that activates additional immune cells, leading to widespread endothelial activation and hemodynamic instability [8,9]. A leading hypothesis for ICANS pathogenesis involves systemic inflammation disrupting the blood–brain barrier (BBB). Elevated cytokines induce endothelial activation in the cerebral vasculature, facilitating immune cell migration into the central nervous system. The resulting neuroinflammation and cerebral edema manifest as neurocognitive symptoms. These risks highlight the critical need for strategies that provide precise spatial and temporal control over CAR activity [10]. Beyond CAR-T cells, CAR-modified natural killer (NK) cells and macrophages represent a promising new frontier. These “off-the-shelf” therapies offer significant advantages, including a potentially better safety profile with reduced toxicity and no need for HLA matching. They also show enhanced efficacy by infiltrating tumors more effectively, targeting cancer cells independent of the often-dysfunctional MHC system, and activating broader anti-tumor immune responses. This makes them particularly compelling for treating solid tumors [11].

Biological nanoparticles (BNPs), such as exosomes, virus-like particles, and biomimetic nanostructures, offer a promising avenue to overcome these limitations [12,13,14,15]. Their inherent biocompatibility and low immunogenicity make them ideal for developing controlled CAR delivery systems that could mitigate risks associated with constitutive expression [12,13]. For instance, BNPs can be engineered for temporary, spatially controlled delivery of CAR-encoding mRNA, potentially preventing the excessive T-cell activation that underlies CRS and ICANS [16].

This review explores modern strategies that leverage BNPs to overcome the limitations of CAR-T cell therapy. The unique properties of BNPs, such as high biocompatibility, low immunogenicity, and targeted delivery capabilities, enable novel approaches for enhancing both the safety and efficacy of CAR-T treatment. The key applications discussed include the non-viral delivery of genetic constructs, the precise spatial and temporal control of CAR-T cell activity, modulation of the immunosuppressive tumor microenvironment, and the development of platforms for in vivo CAR-T cell generation, which could dramatically simplify the therapy and improve its accessibility.

## 2. The Structure of the CAR and Its Evolution

The chimeric antigen receptor (CAR) is a synthetic protein comprising four distinct domains: an extracellular antigen-binding domain, a hinge region, a transmembrane domain, and one or more intracellular signaling domains [15].

**(a)**  Extracellular Antigen-Binding Domain

This domain, typically a single-chain variable fragment (scFv) derived from a monoclonal antibody, confers specificity by recognizing target antigens on cancer cells. The scFv is engineered by linking the variable regions of the heavy (VH) and light (VL) antibody chains with a short peptide linker. A critical functional advantage of the scFv over the native T-cell receptor (TCR) is its ability to bind antigen independently of major histocompatibility complex (MHC) molecules, enabling recognition of a broader range of tumor targets [2,17].

**(b)**  Hinge Region (Spacer)

Acting as a structural adapter, the hinge region is located between the antigen-binding and transmembrane domains. Often derived from immunoglobulin constant regions (e.g., IgG4) or proteins like CD8 or CD28, it provides necessary flexibility. This allows the antigen-binding domain to overcome spatial barriers like the tumor glycocalyx and engage its target effectively. The length and composition of the hinge significantly influence CAR function by modulating binding avidity and immunological synapse formation [10].

**(c)**  Transmembrane Domain

This hydrophobic α-helical domain anchors the CAR within the T-cell membrane. It is commonly borrowed from proteins such as CD3ζ, CD4, CD8α, or CD28. The choice of transmembrane domain affects receptor stability and its interactions with endogenous signaling complexes. For instance, a CD3ζ-derived domain can promote homodimerization with native TCR components, potentially influencing signal strength [10].

**(d)**  Intracellular Signaling Domains

These domains transduce the antigen-binding event into intracellular signals that activate T-cell effector functions, including cytokine production, proliferation, and cytotoxicity. The number and combination of signaling motifs (e.g., from CD3ζ, CD28, or 4-1BB) define the CAR’s generation and functional characteristics [18], as detailed in Table 1.

**Figure 1:**
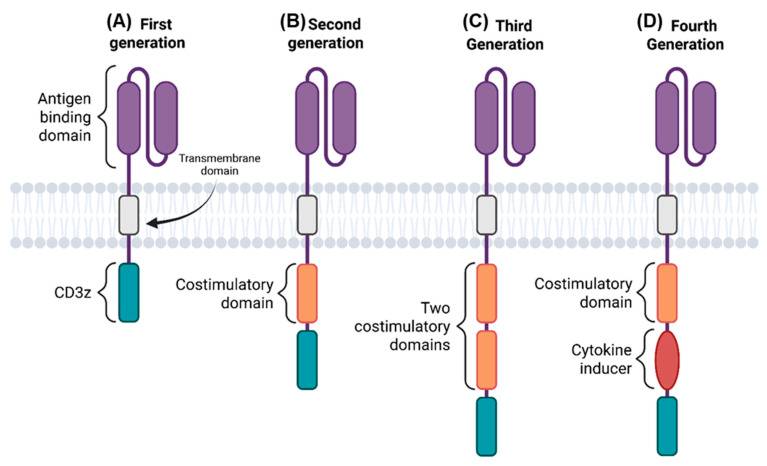
CAR-T generations. (**A**) First generation of CAR consists of extracellular antigen binding domain, transmembrane domain and CD3ζ signaling domain. (**B**) Second generation of CAR has a costimulatory domain in addition to CD3ζ signaling domain. Costimulatory domain is often represented by CD28 or 4-1BB. (**C**) Third generation utilizes two costimulatory domains. (**D**) Fourth generation of CAR utilizes additional modalities, such as cytokine inducers, in addition to costimulatory domain.

First-generation CARs (Figure 1A) incorporate only the CD3ζ signaling domain, which provides a primary activation signal via its immunoreceptor tyrosine-based activation motifs (ITAMs). However, this signal alone is often insufficient to induce robust T-cell proliferation and long-term persistence in vivo [18,19,20].

Second-generation CARs (Figure 1B), which form the basis of most approved therapies, augment the CD3ζ domain with a single co-stimulatory domain. This design provides the necessary dual-signal activation for a full T-cell response: Signal 1 from CD3ζ and Signal 2 from the co-stimulatory domain. This combination significantly enhances proliferative capacity, cytokine production, cytotoxicity, and long-term persistence. The most common co-stimulatory domains, CD28 and 4-1BB (CD137), impart distinct functional characteristics; CD28 domains often promote potent short-term effector responses, whereas 4-1BB domains may favor enhanced persistence and reduced T-cell exhaustion [18,19,20].

Third-generation CARs (Figure 1C) contain the CD3ζ domain plus two different co-stimulatory domains (e.g., CD28 and 4-1BB). While designed to further amplify signaling, these constructs can lead to excessive T-cell activation and subsequent exhaustion, limiting their clinical translation [18,19,20].

Fourth and subsequent generations (Figure 1D), often termed “smart” CARs or TRUCK cells, represent more sophisticated platforms. These designs may incorporate inducible cytokine expression cassettes (e.g., IL-12) to modulate the tumor microenvironment or integrate logic-gate systems (AND, OR) that require recognition of multiple antigens for activation, thereby improving specificity and safety [18,19,20].

## 3. Biological Nanoparticles: Classification and Properties

BNPs can enhance therapeutic efficacy and reduce side effects by delivering effector molecules (drugs or biologics) directly to target cells, such as tumors.

### 3.1. Extracellular Vesicles

The classification of extracellular vesicles (EVs) has traditionally been based on criteria such as their cellular origin, size distribution, and molecular composition. According to these parameters, three main categories are commonly distinguished: exosomes, microvesicles, and apoptotic bodies [21,22]. However, recent evidence highlights significant heterogeneity and overlapping characteristics among these subtypes, underscoring the need for a more comprehensive approach to their identification [23,24].

Exosomes (30–150 nm in diameter) are formed via the endosomal pathway. Their biogenesis begins with the invagination of the membrane of early endosomes, followed by the formation of multivesicular bodies (MVBs) containing intraluminal vesicles. The release of exosomes occurs through the fusion of MVBs with the plasma membrane [21,24,25,26]. Their key advantages over synthetic nanoparticles include innate biocompatibility, low immunogenicity, and a natural capacity for intercellular transfer of functional genetic material (e.g., mRNA, miRNA). The efficacy of EVs is largely determined by their surface molecular composition. The membrane is enriched with tetraspanins (CD9, CD63, CD81), which facilitate cell adhesion and fusion, and integrins that confer tissue-specific tropism [27]. For instance, exosomes expressing integrin αLβ2 (LFA-1) show high affinity for ICAM-1 on activated T lymphocytes, enabling selective targeting [28]. Cargo delivery occurs through ligand-receptor interactions followed by direct membrane fusion or endocytosis, a process that often bypasses lysosomal degradation and ensures efficient cytoplasmic delivery of nucleic acids [29].

To further enhance delivery specificity to T cells, exosome surfaces can be functionalized with ligands that target T-lymphocyte receptors. A common strategy involves genetically engineering producer cells to express chimeric constructs where an EV membrane protein (e.g., Lamp2b) is fused to a targeting ligand, such as an anti-CD3 single-chain variable fragment (scFv). These engineered EVs exhibit significantly higher uptake efficiency by T lymphocytes in vitro and in vivo compared to their native counterpartsn [30].

A major challenge in their purification is the lack of absolutely specific markers, leading to overlapping populations with microvesicles [23,24].

Microvesicles (MVs), also referred to as ectosomes or shedding vesicles, represent a distinct class of extracellular vesicles that are actively released from the plasma membrane through a process of outward budding and fission. Their biogenesis is an energy-dependent mechanism initiated by the activation of enzymes such as flippases and scramblases, leading to a calcium-dependent loss of membrane asymmetry and externalization of phosphatidylserine. This is followed by contraction of the actin-myosin cytoskeleton, ultimately resulting in the pinching-off and release of MVs ranging from 100 to 1000 nm in diameter [31].

The biological significance of MVs lies in their role as crucial mediators of intercellular communication, particularly in the context of cancer. Tumor-derived MVs are not merely passive byproducts but serve as active carriers of diverse molecular cargo selectively enriched from the parent cell. This cargo includes oncogenic proteins (e.g., EGFRvIII), bioactive lipids, mRNAs, microRNAs, and other non-coding RNAs [31]. Upon transfer to recipient cells, this cargo can induce profound phenotypic changes. For instance, in glioblastoma, MVs derived from tumor cells have been shown to deliver oncogenic receptors and microRNAs to less aggressive cancer cells, thereby enhancing their proliferative and invasive potential [31]. Similarly, in hematological malignancies such as leukemia, MVs contribute to the formation of a pro-tumorigenic niche by suppressing the anti-cancer activity of immune cells and promoting angiogenesis and stromal remodeling in the bone marrow [32]. This horizontal transfer of biological information renders MVs instrumental in tumor progression, metastasis, and the development of therapy resistance.

The unique properties of tumor-derived MVs also underpin their substantial therapeutic potential, which can be leveraged through two primary strategies: as biomarkers and as engineered therapeutic vehicles. Their presence in easily accessible biofluids such as blood and their reflection of the parental tumor’s molecular signature make them excellent tools for minimally invasive liquid biopsy in cancer diagnosis, prognosis, and monitoring treatment response [31]. Furthermore, their innate capacity to carry functional biomolecules and target specific cell types opens possibilities for engineering MVs as targeted drug delivery systems. By loading MVs with chemotherapeutic agents, small interfering RNAs, or other therapeutic molecules, their natural tissue tropism can be harnessed for targeted therapy, thereby minimizing off-target effects [32]. Studies in leukemic models have demonstrated the feasibility of using microvesicles (MVs) for the delivery of anti-tumor microRNAs or chemical inhibitors into leukemic cells [32].

Apoptotic bodies (1–5 μm) (ApoBDs), long considered terminal waste products of programmed cell death, are now recognized in modern research as complex extracellular vesicles that play key roles in intercellular communication and the maintenance of homeostasis [33]. Their formation is an active and regulated process initiated by caspases, which cleave structural cytoskeletal proteins, leading to membrane blebbing and the subsequent budding of vesicles containing nuclear fragments, cytoplasm, and intact organelles [34]. A critical aspect of their biogenesis is the active and selective packaging of a specific molecular cargo, including nucleic acids (genomic DNA, microRNAs), proteins, and lipids, which transforms ApoBDs from inert particles into potent signaling modules [33,34].

A central biological function of apoptotic bodies is to facilitate “cellular transfer”—the efferocytosis-mediated delivery of biological materials from the dying cell to the phagocytosing cell [33]. Efficient efferocytosis, triggered by the expression of “eat-me” signals such as phosphatidylserine on the ApoBD surface, not only prevents inflammatory responses but also actively induces the production of anti-inflammatory cytokines, thereby maintaining immune homeostasis [34]. However, this same physiological function can be subverted in pathological conditions. For instance, in oncology, ApoBDs derived from tumor cells can serve as vectors for the horizontal transfer of oncogenic materials (e.g., oncogenic DNA and microRNAs) between cells, potentially enhancing tumor heterogeneity, phenotypic aggressiveness, and therapy resistance [33]. Furthermore, impaired clearance of apoptotic bodies is implicated in the pathogenesis of autoimmune diseases, where the accumulation of non-phagocytosed ApoBDs leads to the presentation of self-antigens and the development of systemic inflammation [34].

(a) Therapeutic Potential: The unique properties of ApoBDs open promising avenues for their use as natural, biocompatible platforms for the targeted delivery of therapeutic agents. Their innate ability to be efficiently taken up by various cell types, modulate immune responses, and carry protected cargo makes them ideal candidates for novel drug development [33]. Potential application strategies include:
(b) Targeted Drug Delivery: Loading ApoBDs with chemotherapeutic agents, anti-cancer RNAs (e.g., siRNA), or immunomodulators allows for the exploitation of their natural uptake mechanism for targeted delivery to specific cells, such as macrophages or tumor cells, thereby enhancing therapeutic efficacy and reducing systemic toxicity [33].
(c) Immunotherapy and Vaccines: Due to their capacity to carry a full spectrum of tumor-associated antigens from the parent cell, ApoBDs can be utilized to develop therapeutic anti-cancer vaccines, enhancing antigen presentation by dendritic cells and stimulating a specific T-cell-mediated immune response against the malignancy [33,34].
(d) Regenerative Medicine: The ability of ApoBDs to transfer signaling molecules and organelles could be harnessed to stimulate reparative processes in damaged tissues, for instance, by delivering proliferative signals or mitochondria to restore cellular energy metabolism [33].

Beyond this broad classification, recent advances in isolation techniques have enabled the detailed characterization of EVs and nanoparticles. This has led to the subdivision of EVs into several distinct types, including: small EVs (comprising classical and non-classical exosomes), large EVs (such as microvesicles and large oncosomes), and other particles like ARMMs, apoptotic bodies, autophagic EVs, and non-vesicular fractions such as exomeres and vault complexes. While these EV subtypes are distinguished by specific protein biomarkers, size, and biogenesis pathways, their biological functions and cargo content remain poorly understood [35,36].

### 3.2. Biomimetic Nanoparticles

**Figure 2:**
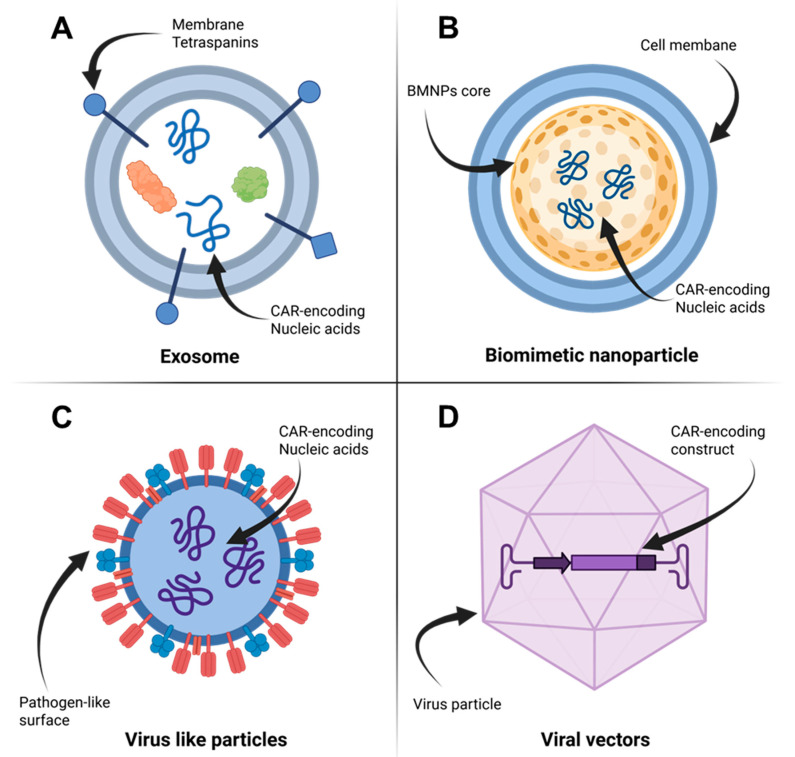
Nanoplatforms for enhancing CAR-T cell therapy. Key nanoparticle types include: (**A**) Exosomes: Naturally secreted biological vesicles with high biocompatibility. Their surface is enriched with tetraspanins for cellular uptake, and they can be engineered to carry specific therapeutic cargo. (**B**) Biomimetic nanoparticles: Synthetic particles consisting of a biocompatible core (e.g., polymer, gold) coated with a membrane derived from cells like erythrocytes or platelets, combining synthetic versatility with natural biointerfacing properties. (**C**) Virus-like particles (VLPs): Self-assembled nanostructures from viral proteins that mimic viral morphology and cell-entry capabilities, capable of being loaded with diverse cargo. (**D**) Viral vectors: Engineered viruses (e.g., AAV, lentivirus) where the native genome is replaced by therapeutic gene cassettes, enabling efficient gene delivery.

Biomimetic nanoparticles (BMNPs) represent a sophisticated class of hybrid nanostructures engineered according to the core–shell principle. These innovative systems integrate a synthetic core, functioning as a cargo carrier, with a biological membrane shell that provides a native interface for optimal interaction with the physiological environment (Figure 2B). This advanced design strategy effectively addresses critical limitations of conventional synthetic nanocarriers—including rapid clearance by the mononuclear phagocyte system (MPS), insufficient target specificity, and inherent immunogenicity. The fundamental concept leverages structural and functional elements from natural entities to emulate their biological behavior, thereby enhancing biodistribution, biocompatibility, and therapeutic efficacy [26,27].

#### Core Materials Strategic: Selection and Function Characteristic

The synthetic core constitutes a fundamental structural element of BMNPs, determining essential characteristics including loading capacity, release kinetics, pharmacokinetic parameters, and the overall size and morphology of the nanosystem. Material selection is strategically guided by the specific therapeutic application and physicochemical properties of the encapsulated cargo, requiring design optimization [37].

Organic nanomaterials: encompassing polymeric and lipid-based structures, are typically fabricated through self-assembly, nanoprecipitation, or emulsion-solvent evaporation techniques. Poly (lactic-co-glycolic acid) (PLGA) nanoparticles occupy a prominent position among polymeric systems due to their exceptional biocompatibility, controlled biodegradability, regulatory approval status (FDA, EMA), capacity for sustained release, and versatility in encapsulating both hydrophobic and hydrophilic compounds. Notably, research demonstrates that biomimetic membrane coatings significantly modulate drug release kinetics, where increased membrane quantity correlates with extended release profiles. For instance, PLGA nanoparticles loaded with trametinib and camouflaged with T-cell membranes exhibit optimal release characteristics alongside enhanced stability and biocompatibility [38].

Lipid Nanoparticles (LNPs) maintain a leading role in nucleic acid delivery applications. Their architecture incorporates cationic or ionizable lipids that efficiently complex with nucleic acids, providing protection against nuclease degradation while facilitating endosomal escape into the cytoplasmic compartment. The modular nature of LNPs enables incorporation of supplementary components including PEG-lipids to extend circulation half-life and helper lipids to enhance structural integrity [30].

Inorganic nanomaterials: significantly expand BMNP functionality through their unique physicochemical properties. A notable example includes Fe_3_O_4_ nanoparticles co-loaded with sulfasalazine (SAS) and camouflaged with platelet membranes (Fe_3_O_4_-SAS@PLT). This innovative system demonstrates synergistic therapeutic effects through ferroptosis induction combined with immunomodulatory activity. The activation of this iron-dependent cell death pathway not only directly inhibits tumor proliferation but also promotes macrophage polarization from immunosuppressive M2 to antitumor M1 phenotypes, substantially enhancing the efficacy of anti-PD-1 immunotherapy [39].

### 3.3. Biomimetic Shells: Biological Targeting Mechanisms

The biomimetic shell is the key functional component of a BMNP. This shell consists of a membrane or its derivatives isolated from specific donor cells, which confers the nanoparticle with the surface properties of the source cell [40].

(1) Red Blood Cell Membranes. This most common type of biomimetic shell confers long circulation times and enhanced tumor penetration. These properties are largely attributed to surface markers like CD47, which binds to the SIRPα receptor on macrophages to inhibit phagocytosis [40].
(2) Platelet Membranes. This type contains surface proteins that promote targeted accumulation at sites of tumor neovascularization. Key mediators include adhesive molecules like P-selectin, which binds specifically to CD44 receptors on tumor cells. Other significant components are the CD40L ligand (involved in immune activation), integrins (CD41, CD61), and glycoproteins (CD42b) [41].
(3) Leukocyte Membranes (e.g., neutrophils, macrophages). Membranes derived from leukocytes possess chemokine receptors that are recruited to pathological tissues by inflammatory signals. Surface receptors such as LFA-1 and Mac-1 facilitate specific binding to adhesion molecules (e.g., VCAM-1) on inflamed endothelium and tumor cells, enabling targeted nanoparticle delivery to diseased sites [41].
(4) Cancer Cell Membranes. This type of membranes contains a unique profile of tumor-associated antigens (TAAs) and adhesion proteins (e.g., EpCAM, integrins) that serve as a tumor-specific “identification signature” [42]. When used to coat synthetic nanoparticles, these membranes create biomimetic systems capable of homotypic targeting: the retained surface proteins mediate specific binding to other cancer cells of the same type through natural cell-adhesion mechanisms. This approach promotes efficient tumor accumulation while simultaneously camouflaging the nanoparticle from immune recognition [8].

### 3.4. Virus-like Particles

Virus-like particles (VLPs) are nanostructures (20–200 nm) formed by the self-assembly of viral structural proteins, which mimic the size and morphology of native virions (Figure 2C). A key characteristic of VLPs is their lack of viral genetic material, which prevents replication while retaining high immunogenicity due to their pathogen-like surface patterns recognizable by the immune system [30,31]. This combination of safety and strong immunogenicity makes VLPs highly suitable for vaccine development [43,44,45].

VLPs can be derived from various viruses. A prominent example is the vesicular stomatitis virus (VSV). Recombinant VSV-based VLPs (VSV-VLPs) can be engineered to express heterologous viral glycoproteins, accurately mimicking the antigenic landscape of target pathogens without containing the viral genome [46]. Their structure is formed by the VSV matrix protein (M-protein), which interacts with the host cell lipid membrane [47]. The key surface component is the glycoprotein (G-protein), which mediates receptor recognition on target cells [34,35]. Due to its modular nature, the G-protein can be replaced with glycoproteins from other viruses (e.g., VSV-EBOV), enabling the creation of chimeric VLPs with tailored antigenic properties [48].

- Hepatitis B Surface Antigen (HBsAg) VLPs

VLPs based on the hepatitis B surface antigen (VLP-HBsAg) are recombinant nanoparticles morphologically and antigenically identical to native subviral particles from infected individuals [49]. Their structure consists of a lipid bilayer embedded with numerous HBsAg monomers [50]. This platform has a well-established role in vaccinology; while early hepatitis B vaccines used particles purified from patient plasma, later generations employed recombinant HBsAg produced in yeast or mammalian cells to ensure complete safety [43]. However, due to universal use anti-HBV vaccination, such VLPs will be effective only for ex vivo application. Strong antiviral immunity would prevent such VLPs to function in vivo.

- Adeno-Associated Virus (AAV) VLPs

The capsid proteins of adeno-associated virus (AAV), a common gene therapy vector, can form VLPs. These structures hold potential for efficient DNA cargo delivery, leveraging AAV’s natural tropism and low immunogenicity [51].

- Bacteriophage VLPs

Capsid proteins from bacteriophages can be engineered into highly stable VLPs. These nanoparticles have promising applications in vaccinology and as nanocarriers for drug delivery due to their robustness and modularity [52].

Structurally, VLPs are classified into two main types:

1. VLPs without a lipid envelope: Composed exclusively of capsid proteins [51].
2. VLPs with an envelope: Incorporating a lipid membrane derived from the host cell during the assembly and budding process [51].

VLPs can assemble into single-layer, double-layer, or multi-layer architectures. While traditional VLPs are composed of proteins from a single virus, chimeric VLPs can be engineered through the co-assembly of structural proteins derived from different viruses. The VLP’s virion-mimetic structure facilitates efficient recognition and uptake by target cells. Following internalization, the particle is transported to the cytosol where it disassembles, releasing its therapeutic payload. A key advantage of VLPs is their versatility as a delivery platform. The internal cavity functions as a nanocontainer for encapsulating diverse cargo, such as nucleic acids, peptides, proteins, and small molecules. For targeted protein delivery, genetic engineering can be used to fuse the protein of interest to a VLP structural protein via a protease-cleavable linker. This allows for controlled release within the target cell or nucleus upon cleavage by a co-delivered viral protease or endogenous cellular mechanisms [51].

## 4. The Use of BNPs in CAR-T Therapy

The production of CAR-T cells is a complex, multi-step process requiring meticulous monitoring. The two primary manufacturing strategies are: (1) the creation of allogeneic, “off-the-shelf” products from healthy donors, and (2) the patient-specific autologous approach, which involves harvesting, engineering, and reinfusing the patient’s own T cells [53,54].

A fundamental distinction in CAR-T manufacturing is the locus of genetic modification, whether it occurs ex vivo or in vivo, as this dictates the entire logistical framework, complexity, and risk profile of the treatment [53,54].

The main stages of the ex vivo modification process include: isolation of T cells from the patient’s peripheral blood, their genetic modification for expression of the CAR, activation and expansion in vitro, as well as preparation for therapeutic infusion. The effectiveness and safety of the final product depend on the CAR gene delivery methods, T cell stimulation methods, and the types of vectors used [41,42].

(a) The initial stage is the collection of peripheral blood mononuclear cells (PBMC) of the patient by leukocyte apheresis, which allows selectively isolating the leukocyte fraction with minimal loss of cellular material. Then selective isolation of T-lymphocytes is carried out [55].
(b) The key stage of CAR-T therapy is the introduction of the CAR gene into the genome of the patient’s T-lymphocytes, using vector transduction based on viral or non-viral systems. The most common vectors for transduction are those based on AAV or lentiviruses. After transduction, successfully modified cells are selected and then activated in vitro [56].

Recent advances in genetic engineering have demonstrated the significant potential of combining adeno-associated virus (AAV) with the CRISPR/Cas system for CAR-T cell generation. This platform enables targeted integration of the CAR gene while simultaneously knocking out undesirable genes, facilitating the development of more potent and versatile “off-the-shelf” therapeutic products. Specifically, AAV delivers a template for precise insertion of the CAR gene into defined genomic loci such as the TRAC locus (T cell receptor alpha constant chain gene). This targeted integration strategy addresses two major challenges in CAR-T therapy: it ensures unified CAR expression under the control of the endogenous promoter, resulting in more uniform and physiologically regulated receptor expression, and it enhances cellular efficacy, with such “enhanced” CAR-T cells demonstrating superior antitumor activity compared to cells generated via random integration. Furthermore, the AAV-CRISPR/Cas platform allows for concurrent knockout of endogenous genes to improve CAR-T cell functionality. Key knockout targets include immune checkpoint genes (e.g., PD-1), where CRISPR/Cas-mediated knockout prevents interaction with the PD-L1 ligand on tumor cells, thereby blocking a major mechanism of antitumor response suppression; and genes associated with graft rejection (e.g., TCR, B2M), where knockout of the native T-cell receptor enables generation of allogeneic CAR-T cells from donor cells while minimizing graft-versus-host disease risk, and knockout of beta-2-microglobulin eliminates MHC class I expression, reducing immune recognition and elimination of allogeneic CAR-T cells by the recipient’s immune system [57]. Recent advances have introduced exosome-based gene editing tools capable of efficiently delivering short-lived CRISPR/Cas9 ribonucleoprotein (RNP) complexes. This approach enables scarless, traceless genome editing by eliminating the sustained presence of CRISPR components [58,59,60].

(a) Activation is aimed at inducing proliferation and enhancing the efficiency of genetic modification. In vitro, physiological activation mediated by TCR and co-stimulatory signals (e.g., via CD28) is simulated using artificial stimuli. The most common method involves the use of magnetic nanoparticles conjugated with antibodies against CD3 and CD28 [61]. Additionally, cytokines (such as IL-2, IL-7, and IL-15) are employed to stimulate T cells [62].
(b) Genetically modified CAR-T cells are administered to the patient intravenously as a slow infusion. Before the infusion of CART cells to the patient, lymphodepletion is performed, which helps to reduce the cytotoxic response of the body and provides favorable conditions for the proliferation of CAR-T cells [63].

The primary advantage of this approach is the complete control over the modification process and the quality of the final cellular product. However, this method is associated with several drawbacks: an extended production timeline (spanning several weeks), high manufacturing costs, and the inability to treat patients with severe lymphopenia. Furthermore, it is associated with considerable adverse effects, such as immune-mediated complications (e.g., graft-versus-host disease and fratricide in allogeneic settings), cytokine release syndrome, and neurotoxicity. Additional challenges encompass limited efficacy against solid tumors, the development of treatment resistance leading to disease relapse, and the difficulty in achieving long-term persistence of CAR-T cells in vivo [56].

**Figure 3:**
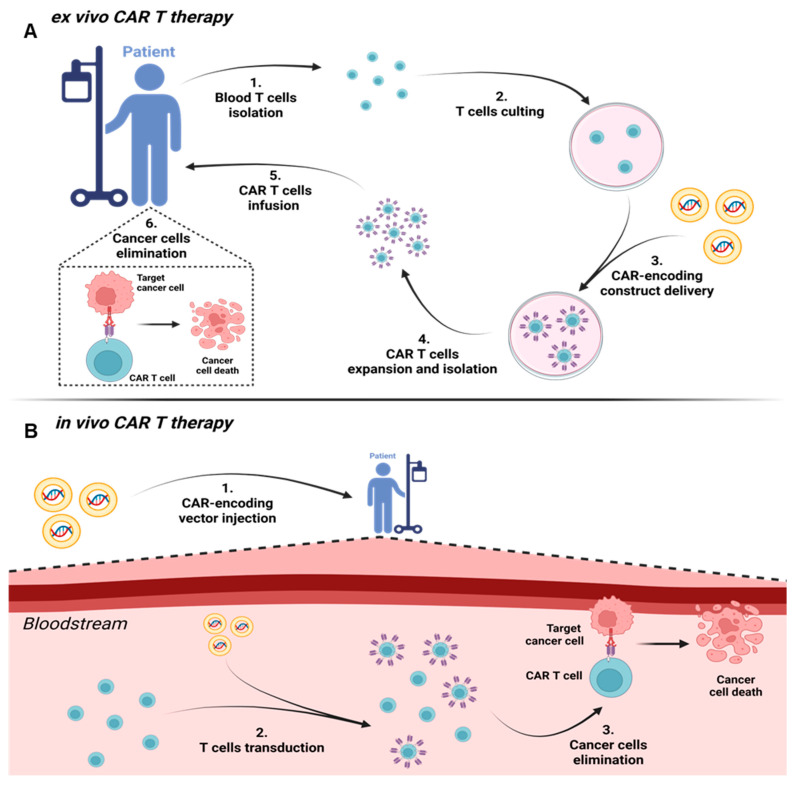
Comparative Overview of Ex Vivo and In Vivo CAR-T Cell Production. **(A) Ex Vivo CAR-T Cell Therapy (Top Panel)** The ex vivo pathway involves the external genetic modification and expansion of patient-derived T-cells before reinfusion. **(1) Blood Cell Isolation:** Peripheral blood mononuclear cells (PBMCs) are collected from the patient via leukapheresis. T-lymphocytes are subsequently isolated from the PBMC fraction. **(2) T Cell Culturing:** The isolated T-cells are activated and cultured in vitro under controlled conditions, preparing them for genetic engineering. **(3) CAR-Encoding Construct Delivery:** The T-cells are genetically modified through transduction (typically using viral vectors like lentivirus or gamma-retrovirus) or transfection (e.g., with mRNA or non-viral nanoparticles) to introduce the CAR gene. The CAR construct enables the T-cells to recognize a specific tumor-associated antigen. **(4) CAR-T Cells Expansion and Isolation:** Successfully transduced CAR-T cells are selectively expanded in culture to achieve a therapeutically relevant cell number. The final product undergoes quality control checks before harvest. **(5) CAR-T Cells Infusion:** The expanded and validated CAR-T cell product is infused back into the patient, often following a lymphodepleting chemotherapy regimen to enhance engraftment and efficacy. **(6) Cancer Cells Elimination:** The infused CAR-T cells circulate, recognize, and bind to target cancer cells via their CAR, initiating a cytotoxic immune response that leads to cancer cell death (e.g., via perforin/granzyme release and cytokine signaling). **(B) In Vivo CAR-T Cell Therapy (Bottom Panel)** The in vivo pathway aims to generate CAR-T cells directly within the patient’s body, bypassing complex external manufacturing. **(1) CAR-Encoding Vector Injection:** A targeted delivery vehicle (e.g., an engineered viral vector like AAV, or non-viral lipid nanoparticles (LNPs) containing CAR-encoding mRNA/DNA) is administered systemically to the patient. **(2) In Bloodstream: T-Cells Transduction:** The circulating vectors/particles specifically target and transduce T-cells within the patient’s lymphoid organs and bloodstream. The genetically reprogrammed T-cells then begin to express the CAR on their surface and expand in situ. **(3) Cancer Cells Elimination:** The newly formed CAR-T cells, generated directly in the body, traffic to tumor sites, recognize the target antigen, and mediate the destruction of cancer cells.

Thus, the multi-stage process of ex vivo modification and activation of T-cells is fundamentally distinct from the promising strategy of generating CAR-T cells in vivo, where vector systems are delivered directly to the patient’s lymphoid organs. To provide a clear comparison of these two fundamentally different approaches—the complex, multi-stage ex vivo manufacturing and the potentially more streamlined and versatile in vivo method—Figure 3 presents a schematic diagram that illustrates their key logistical and technical differences.

In the search for more efficient and versatile platforms capable of overcoming the limitations of existing approaches, researchers have turned their attention to BNPs, owing to their unique biocompatible properties and multifunctionality. BNPs can be used as highly efficient and safe conditions for the generation of CAR-T cells in vivo and ex vivo, as well as an activator and inhibitor of CAR-T cells [48,49,50,51,64].

A compelling application of BNPs involves the creation of cell-mimetic nanocarriers that combine the targeting specificity of CAR-T cells with therapeutic payloads. For instance, one study focused on developing CAR-T cell membrane-coated nanoparticles (CIMs) specifically designed for targeted therapy of hepatocellular carcinoma (HCC), utilizing the expression profile of glypican-3 (GPC3)-a heparan sulfate proteoglycan expressed in 75% of HCC cases but absent in healthy liver tissues. The platform was constructed by first generating GPC3-specific CAR-T cells via lentiviral transduction. The CAR construct contained a GPC3-specific single-chain variable fragment (scFv) derived from GC33, combined with human CD8α hinge domain, CD28 transmembrane domain, and intracellular signaling domains comprising CD28 cytoplasmic domain and CD3ζ molecule. Concurrently, mesoporous silica nanoparticles (MSNs) were synthesized and loaded with IR780 iodide-a near-infrared (NIR) fluorophore with demonstrated photothermal conversion capability. CAR-T cell membranes were then isolated and fused with the IR780-loaded MSNs through a precise extrusion process, yielding the final CIMs. These nanoparticles demonstrated excellent tumor-targeting capability in both in vitro and in vivo HCC models. Under 808 nm laser irradiation, the CIMs generated sufficient thermal energy for effective tumor cell ablation while simultaneously providing real-time guidance through their NIR fluorescence, all while exhibiting low systemic toxicity. The CAR-T cell membrane coating confers specific targeting ability toward GPC3-positive HCC cells, while the MSN core provides efficient photothermal conversion and drug delivery capacity. The preserved orientation of membrane proteins, particularly surface-exposed scFv domains, ensures maintained recognition of tumor-associated antigens, creating a synergistic effect that enhances treatment efficacy while minimizing damage to healthy tissues [65].

### 4.1. In Vivo CAR Therapies

In recent years, several research groups have reported successful in vivo generation of CAR-T cells in mouse models transplanted with human PBMCs. These vectors, often based on AAV or packaged within BNPs, carry the genetic sequence encoding the CAR [48,49,50]. Surface signal peptides enable the vectors to bind specifically to T cells, facilitating cellular entry and delivery of the CAR transgene. This in situ modification has been demonstrated in animal models, where autologous lymphocytes successfully acquired the ability to recognize and eliminate tumor cells [51,52]. Among the various platforms for the delivery of genetic material, viral vectors occupy a leading position due to high efficiency of transduction. In particular, AAV are considered as one of the most suitable options for this task due to the lowest risk of toxicity compared to other viral vectors, ability to infect both dividing and non-dividing cells, and widespread use in clinical practice as a vector for the delivery of therapeutic genes. AAV encoding the CAR gene is able to effectively reprogram immune effector cells in vivo to create functional CAR-T cells, which leads to tumor regression. However, traditional AAV vectors have insufficient tropism (the ability to infect multiple cell types, not just T-lymphocytes) [66].

**Figure 4:**
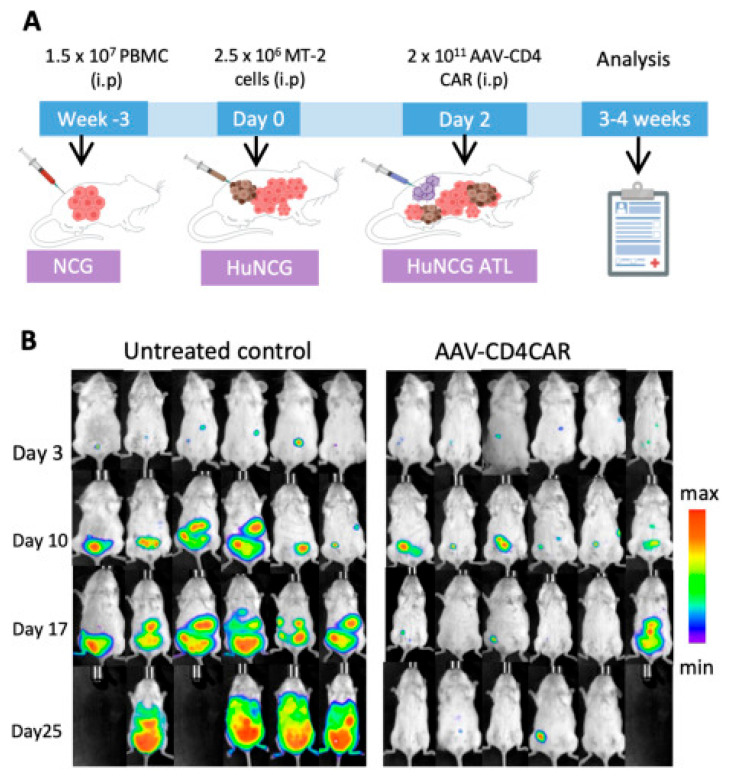
In vivo-generated AAV-CD4CAR T-cell-mediated antitumor activity. (**A**) Schematic representation showing the development of HuNCG ATL mouse model and experimental design. (**B**) Sequential in vivo imaging of luciferase-expressing MT2 ATL cells injected into NCG-HuPBL mice. Following AAV or PBS (untreated) injection, mice were imaged the next day, followed by in vivo imaging at different time points as specified in the figure. Blank images represent dead mice. Figure rearranged and reprinted from [68], licensed under CC BY 4.0.

The therapeutic potential of AAV platform is confirmed in experimental models: encapsidation into AAV of a genetic construct encoding third-generation CAR (with CD28, 4-1BB, and cd3ζsignaling domains) leads to effective generation of functional CAR-T cells directly in vivo. This resulted in significant tumor regression and the establishment of a stable antitumor immune response in the NCG-HuPBL mouse model, which contains 80–90% human T cells (CD45+/CD3+), demonstrating the potential to avoid complex and costly ex vivo cell culture procedures (Figure 4) [67]. The use of biomimetic nanoparticles based on AAV could help overcome the low-level tropism of native viral particles, thereby enhancing the therapy’s efficacy. However, these viral-based methods share the limitations of conventional gene therapies, such as the potential for immunogenicity and restrictions to single-use administration. In this context, non-viral approaches represent a more promising alternative [54,55].

One of the promising directions in the development of in vivo methods for the delivery of genetic constructs for CAR-T cell therapy is the use of non-viral delivery vehicles, such as exosomes or extracellular vesicles. In an exemplar study, an innovative platform based on exosomes produced by dendritic cells stimulated by tumor antigens (tDC-Exo) was developed. These nanoparticles contained major histocompatibility complexes with tumor antigens and co-stimulatory CD86 molecules, which provided the necessary signals for T-cell activation. To enhance targeted interaction between immune and tumor cells, the exosomes were functionalized with bispecific antibodies against CD3 (on T-lymphocytes) and against the epidermal growth factor receptor (EGFR, associated with cancer cells). This dual modification created a “bridge” between the target cell populations, enabling specific binding to tumor cells and efficient activation of endogenous T-lymphocytes to generate a CAR-T-like response directly in vivo. In vitro experiments demonstrated that the functionalized exosomes (Exo-OVA-aCD3/aEGFR) significantly enhanced T-cell proliferation and activation, and mediated their adhesion to cancer cells. In vivo studies confirmed the platform’s pronounced antitumor activity [51].

Recent studies demonstrate that exosomes released ex vivo by CAR-T cells carry functional CAR on their surface, contain cytotoxic molecules (granzymes, perforin), and are capable of specifically lysing target tumor cells in vitro and in vivo [56,57]. An important advantage of CAR exosomes over cell therapy is their enhanced safety profile: they do not express inhibitory receptors such as PD-1, which makes their cytotoxic effect resistant to the immunosuppressive influence of the PD-L1 ligand, and they also do not cause cytokine release syndrome (CRS) in preclinical model [69]. The protocol for obtaining such exosomes involves the isolation of T cells from peripheral blood, their genetic modification using lentiviral vectors to express a second-generation CAR (e.g., based on scFv from cetuximab or trastuzumab, and CD8a, 4-1BB, and CD3ζ domains), activation and stimulation with antigen-expressing cells to enhance the production of CAR-containing exosomes, followed by their purification using ultracentrifugation or affinity capture methods [70].

Beyond artificially engineered exosome platforms, naturally produced CAR-positive EVs (CAR + EVs) represent another important facet of EV biology in CAR-T therapy. CAR-positive EVs (CAR + EVs) are nanoscale particles produced by CAR T-cells in vivo following their infusion into patients. Their key structural characteristic is the presence of a full-length chimeric antigen receptor (CAR) on their external membrane, analogous to the receptor found on the parent CAR T-cells. These vesicles are generated through budding from the membrane of circulating CAR T-lymphocytes and retain not only the CAR (targeting CD19) but also other proteins characteristic of T-lymphocyte surfaces, which allows for the identification of their cellular origin. Like other EVs, CAR + EVs carry intraluminal cytoplasmic components, including proteins, nucleic acids, and signaling molecules, although the primary focus in the clinical context is on the membrane-bound CAR as a biomarker [71].

The clinical significance of CAR + EVs lies in their role as a predictive biomarker for Immune Effector Cell-Associated Neurotoxicity Syndrome (ICANS). The level of CAR + EVs in plasma increases significantly prior to the appearance of clinical symptoms of neurological toxicity, creating a ‘window of opportunity’ for preemptive intervention. Furthermore, the peak concentration of CAR + EVs demonstrates a direct correlation with the maximum severity of the ensuing ICANS, allowing for the prediction of not only the onset of the complication but also an assessment of its potential severity. Although the primary focus is on their biomarker function, the presence of CAR + EVs in the circulation suggests their potential involvement in the pathogenesis of ICANS, possibly through interaction with CD19-expressing cells of the blood–brain barrier. Real-time monitoring of CAR + EV levels enables the identification of high-risk patients and the timely administration of corticosteroids or other immunosuppressive agents, forming the basis for a precision medicine approach to enhancing the safety profile of CAR-T cell therapy [71].

**Figure 5:**
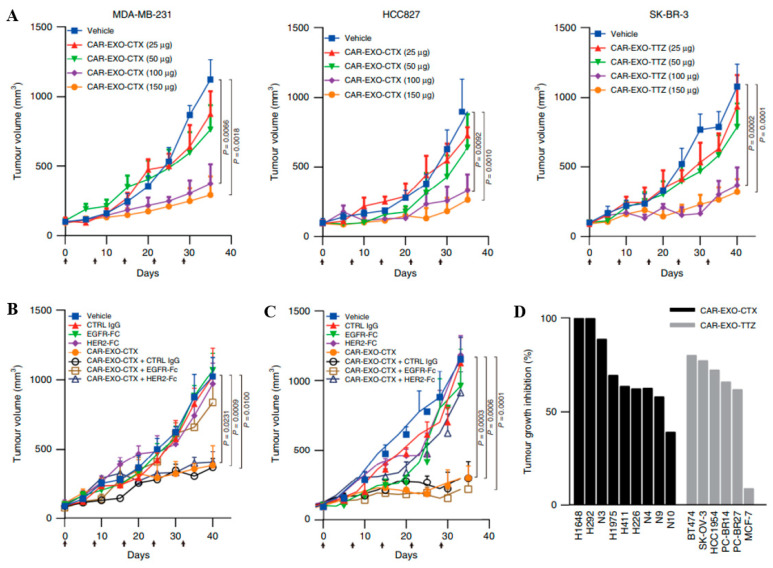
CAR exosomes have notable antitumour activity in vivo. (**A**) Tumor volumes of MDA-MB-231 (**left**), HCC827 (**middle**) and SK-BR-3 (**right**) tumor xenografts after treatment with the indicated treatment, *n* = 8. (**B**,**C**) Tumor volumes of MDA-MB-231 (**B**) and SK-BR-3 (**C**) tumor xenografts after treatment with the indicated CAR exosome treatment with or without blocking recombinant antigen, *n* = 8. (**D**) Cancer cell lines or patient-derived tumor tissue fragments established as subcutaneous xenografts (*n* = 8) and treated with weekly doses of CAR exosomes (100 μg). Substantial TGI was observed in lung cancer models treated with CAR-EXO-CTX (black bars) and in HER2-positive breast and ovary cancer models treated with CAR-EXO-TTZ (gray bars). Arrows indicate the treatment point (**A**–**C**). Data are means ± s.e.m. (**A**–**C**). *p* values are from a two-way ANOVA followed by Bonferroni post-test (**A**–**C**). Source data (**A**–**D**) are provided as a Source Data file. This figure is derived from the source from [70], licensed under CC BY 4.0.

**Figure 6:**
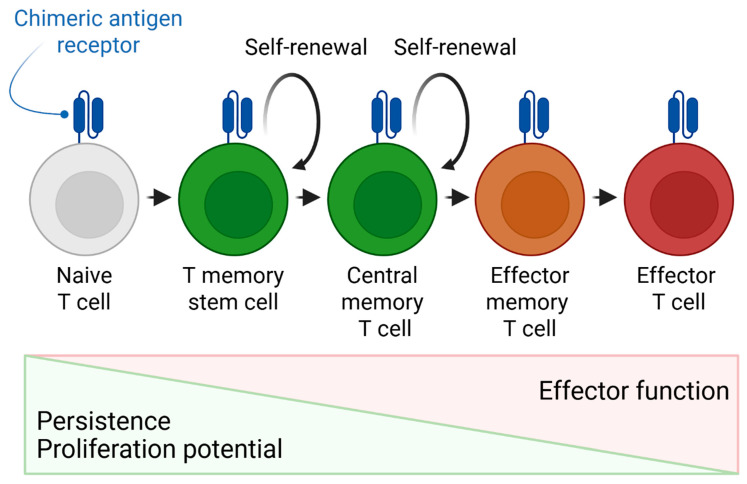
Linear model of T cell differentiation from T naive cells to effector T cells in CAR-T technology. The effectiveness of cell therapy directly depends on the ability of the administered CAR-T cells to persist long-term and self-renew in the patient’s body. T Naïve cells (TN), T Memory Stem Cell (TSCM) cells, and Central Memory T cells (TCM), which are capable of multiple divisions and maintain a pool of effector cells, provide long-term tumor control and prevent relapse, have the greatest therapeutic potential. Strategies are aimed at enriching the production of these self-reproducing populations.

The key task of CAR-T therapy development is to obtain a population with a predominance of memory stem cells (Tscm) with the highest proliferative capacity and persistence in vivo (Figure 5). Exosomes secreted by mesenchymal stromal cells (MSCs) or dendritic cells (DCs) carrying immunomodulatory signals (cytokines, chemokines, miRNAs) can be used as adjuvants in a culture medium. For example, exosomes containing IL-15 or IL-21 may contribute to the maintenance of the memory phenotype, while particles with TGF-β may inhibit differentiation into undesirable effector subpopulations [72]. To conclude, exosomes are perspective delivery vehicles for CAR genetic constructs, especially in light of technological advances in their manufacturing and development of elaborated methods for packaging and delivering genetic constructs (Figure 6) [59,60,61]. Furthermore, their nanoscale structure and innate ability to traverse biological barriers confer superior permeability into the dense stromal tissue of solid malignancies compared to bulkier CAR-T cells, making them a particularly promising technology for solid tumor therapy [73]. Their mechanism of action can be further augmented by engineering exosomes to deliver not only cytotoxic signals but also immunomodulatory molecules (e.g., cytokines or chemokines) capable of disrupting the immunosuppressive tumor microenvironment, thereby overcoming a major barrier in cellular immunotherapy [73]. The biological foundation for such intercellular communication, involving the transfer of membrane receptors, has been established in fundamental research [74].

#### Challenges and Limitations of In Vivo CAR-T Cell Generation

Notwithstanding the considerable progress in developing platforms for the in vivo generation of CAR-T cells, this innovative approach is confronted by several significant challenges that must be addressed to ensure its successful clinical translation. A primary limitation of viral vectors, notably adeno-associated viruses (AAV), is their insufficient specificity (tropism) for T lymphocytes. This promiscuity can lead to the transduction of off-target cell types, potentially reducing therapeutic efficacy and increasing the risk of adverse effects [75]. Furthermore, the immunogenicity of viral vectors remains a substantial barrier, which can preclude repeated administration and carries a risk of severe adverse events [76,77,78].

Concerning non-viral approaches, such as those utilizing exosomes, the principal challenges encompass achieving high-efficiency delivery of genetic cargo to the nucleus of T cells and standardizing protocols for the isolation, loading, and purification of these nanovesicles to ensure batch-to-batch reproducibility and scalable manufacturing [12,53,58,69,79]. Although CAR-bearing exosomes exhibit a superior safety profile—including the absence of cytokine release syndrome (CRS) and resistance to the immunosuppressive tumor microenvironment (e.g., via the PD-1/PD-L1 axis) [69]—issues pertaining to their precise targeting, in vivo stability, and the long-term persistence of the antitumor response necessitate further investigation [53].

Consequently, while both viral and non-viral in vivo platforms demonstrate remarkable therapeutic potential in preclinical models, overcoming these delineated limitations is a critical prerequisite for their subsequent advancement into clinical applications.

### 4.2. Ex Vivo and In Vivo CAR Cell Engineering: Comparative Challenges and Delivery Platforms

The development of in vivo CAR therapies is motivated by several limitations of ex vivo approaches, including their significant cost, highly complex manufacturing, and potential for reduced efficacy. A key hypothesis is that in vivo-generated CAR cells, derived from a non-exhausted, intact immune system, may be more potent fighters against cancer than the ex vivo-modified. Moreover, the required lymphodepletion chemotherapy prior to CAR cell infusion can impair the patient’s broader, innate anti-cancer immune responses. However, this very advantage presents a challenge: an intact immune system may mount a response against the engineered cells or delivery vehicle. Consequently, the need for at least partial lymphodepletion in in vivo approaches is being re-evaluated based on early clinical data [74].

A primary safety concern for in vivo CAR therapy is the risk of off-tissue targeting and insertional oncogenesis, leading to secondary cancers [80] or technological pitfalls that compromise the efficiency of CAR therapies [81]. This risk is predominantly associated with integrating viral vectors (e.g., lentiviruses). In contrast, non-viral platforms, which typically enable transient gene expression, offer a safer profile in this regard [78]. The choice of delivery platform is thus critical. The landscape ranges from traditional viral vectors (lentivirus, AAV) to non-viral methods employing DNA or mRNA.

Among non-viral options, mRNA-loaded lipid nanoparticles (LNPs) are versatile but confer very transient CAR expression (often <1 week) [82]. While this transient nature can be a safety feature, it may require repeated administrations, which is only feasible with the lack of immunogenicity and an acceptable toxicity profile. Circular RNA (circRNA) represents a promising alternative, capable of sustaining expression for several weeks or months [83], but it faces fundamental challenges in design, production, and achieving high expression levels [84,85]. Despite these hurdles, non-viral methods hold irrefutable advantages in cost, manufacturing complexity, and safety, though their efficiency, targeting precision, and longevity of expression remain key hurdles [86].

The clinical trial landscape reflects this technological divide. To date, over 14 clinical trials are investigating in vivo CARs, with a nearly equal split between viral and non-viral (primarily LNP-based) delivery. Current LNP trials are exploring both mRNA (5 studies) and circRNA (3 studies) for a range of B-cell cancers, solid tumors, and autoimmune diseases. A critical bottleneck is the lack of targeted delivery. Notably, only one of these LNP trials (CPTX2309) has disclosed the use of a targeted LNP, functionalized with an anti-CD8 antibody [74].

Future refinements will depend on enhancing the specificity of these platforms. While current non-viral vehicles are largely limited to LNPs, liposomes, and polymeric nanoparticles, next-generation biomimetic systems like exosomes are particularly promising [87,88,89,90]. Promising results from preclinical studies underscore the translational potential of this approach. Specifically, an innovative study developed an inhalable formulation of exosomes derived from CAR-T cells targeting the epidermal growth factor receptor (EGFR) [91]. Structurally, these therapeutic agents consisted of CAR-T cell-derived exosomes displaying a full-length anti-EGFR CAR on their surface and encapsulated with the chemotherapeutic agent paclitaxel. The mechanism of action of this agent is dual: the surface CAR mediates specific recognition and binding to EGFR-positive tumor cells, while the paclitaxel released into the target cells induces apoptosis by stabilizing microtubules and disrupting cell division. Results obtained in a murine model of non-small cell lung cancer demonstrated that the inhalable delivery of this complex (ExoPAC) led to statistically significant suppression of tumor growth compared to an equivalent dose of free paclitaxel. Furthermore, combination therapy involving ExoPAC and targeted CAR-exosomes (without the chemotherapeutic agent) produced a synergistic effect, resulting in near-complete tumor regression, indicating a potent antigen-specific cytotoxic capacity of the exosomes themselves [91].

Another study focused on the generation and characterization of exosomes carrying a CAR against the classic B-cell antigen CD19 [69]. Structurally, these CAR-exosomes were produced by the HEK293T cell line transfected with a plasmid encoding an anti-CD19 CAR, ensuring the incorporation and surface exposure of the functional receptor. Their mechanism of action involves the direct induction of apoptosis in target cells. The binding of the CAR-exosome to the CD19 antigen on the tumor cell surface initiates intracellular signaling cascades analogous to those triggered by CAR-T cells, ultimately leading to target cell death. In vitro results confirmed that such CAR-exosomes exhibit high affinity and specificity, effectively lysing CD19-positive tumor cells. Crucially, unlike live CAR-T cells, they are incapable of proliferation, fundamentally mitigating the risk of an uncontrolled immune response and CRS development [69].

Their superior biocompatibility and cellular internalization characteristics, coupled with advances in nucleic acid loading, could unlock the potential for highly specific, efficient, and safe in vivo CAR engineering [36,92,93,94].

## 5. Targeted Activation and In Vivo Control

BNPs can act as a “remote control” for T-lymphocytes and other immune cells, operating through both intrinsic genetic engineering and extrinsic nanoparticle-based delivery systems [65,76]. One fundamental approach involves the precise insertion of a CD19-specific CAR construct into the T-cell receptor α constant (TRAC) locus using CRISPR/Cas9-mediated editing coupled with adeno-associated viral (AAV) vectors as delivery vehicles. This strategy prevents TCR mispairing that could cause off-target reactivity, ensures physiological CAR expression levels that minimize tonic signaling and maintains natural feedback mechanisms for controlled activation. The utilization of AAV vectors as nanocarriers demonstrates critical advantages in this context, providing high-efficiency gene delivery while maintaining specificity. When combined with Cas9 ribonucleoprotein complexes, these viral vectors facilitate knock-in efficiencies exceeding 40% with minimal off-target effects. The resulting TRAC-CAR T cells exhibit homogeneous CAR expression, contrasting sharply with the variegated expression patterns observed in conventional retrovirally transduced counterparts. This uniform expression profile proves functionally significant, as it eliminates antigen-independent tonic signaling. Comparative in vivo assessments using stringent leukemia models reveal substantial functional advantages of TRAC-CAR T cells. The precisely controlled CAR expression correlates with enhanced persistence and sustained antitumor activity, particularly at limiting cell doses. Mechanistic investigations attribute this superior performance to reduced terminal differentiation and exhaustion. Furthermore, the endogenous TRAC promoter enables dynamic regulation of CAR surface expression following antigen engagement, characterized by appropriate internalization and subsequent recovery to baseline levels [95].

Contemporary advances in immunotherapy underscore the considerable potential of in vivo T-lymphocyte reprogramming strategies utilizing mRNA delivery via ionizable lipid nanoparticles (mRNA-LNPs). For instance, one research initiative in this domain has established a targeted platform for efficient intracorporeal delivery of mRNA encoding a CAR directly into T cells. The molecular target selected was the TRP1 antigen, which demonstrates high specificity towards melanoma cells. A pivotal component of the developed system involved the directional targeting of nanoparticles to T lymphocytes through conjugation with anti-CD3 antibodies (aCD3), substantially enhancing the selectivity and efficiency of the entire process [96].

The engineered ionizable lipid nanoparticles exhibited a multi-layered architecture. The structural foundation comprised the ionizable lipid SM-102, capable of protonation under the acidic conditions of the endosomal pH, thereby facilitating subsequent content release. Stabilization of the lipid bilayer was achieved by incorporating 1,2-distearoyl-sn-glycero-3-phosphocholine (DSPC) and cholesterol. To minimize premature clearance and confer stealth properties, the PEG-lipid DSPE-PEG2000-Biotin was utilized [5].

The mRNA encapsulated within the nanoparticles encoded a CAR specific to the melanoma-associated antigen TRP1. The receptor design incorporated an scFv fragment derived from the murine monoclonal antibody TA99, a CD8 transmembrane domain, and the intracellular signaling domains CD28 and CD3ζ. To ensure targeting specificity, the nanoparticles were functionalized via streptavidin-biotin binding with anti-CD3 antibodies. In vivo studies confirmed the preferential accumulation of the modified aCD3-LNPs in the spleen, the primary organ for antigen presentation to T lymphocytes [5].

The process of genetic modification of T lymphocytes in vivo using CD3-CAR-LNPs constitutes a sequence of interconnected molecular events. The initial stage involves the specific binding of functionalized nanoparticles to the ε-subunit of the CD3 receptor on the plasma membrane of T cells. This highly selective interaction initiates the internalization process. The subsequent stage entails receptor-mediated endocytosis, during which the ligand-receptor complex invaginates to form clathrin-coated vesicles. This process leads to the incorporation of nanoparticles into membrane-bound compartments—early endosomes. A critical step is endosomal escape, facilitated by the unique properties of the ionizable lipid SM-102. Upon acidification of the endosomal lumen to pH values of approximately 5.0–6.5, protonation of the amine groups in SM-102 occurs, inducing a structural rearrangement of the lipid packing and destabilization of the endosomal membrane. This mechanism ensures the release of the nucleic acid into the cytosol, preventing its lysosomal degradation. Following successful endosomal escape, the mRNA is released into the cytosol, where it becomes accessible to the cellular translational machinery. During the translation and assembly of the CAR on ribosomes, synthesis of the chimeric receptor polypeptide chain occurs. The newly synthesized protein undergoes post-translational modifications, after which the CAR gains functional activity and the capacity for specific antigen recognition. The in vivo-generated mRNA-CAR-T cells demonstrated a range of functional characteristics confirming their therapeutic efficacy. The conducted cytotoxicity assays established that the genetically modified lymphocytes exhibited selective cytolysis of TRP1-positive B16F10 melanoma cells while showing no effect on TRP1-negative cell lines. CAR activation induced intense secretion of effector cytokines (IFN-γ, TNF-α), indicative of the full functional competence of the modified cells [5].

A crucial feature of the platform was the transient expression of the CAR, with peak protein expression observed at 1–2 days post-transfection, followed by a decline to minimal levels by day 7. This kinetic profile, attributable to the metabolic degradation of mRNA, ensured the absence of signs of acute toxicity and cytokine release syndrome (CRS) [5].

To overcome the immunosuppressive tumor microenvironment, a combined therapeutic strategy was developed. Within this framework, CD3-7CAR-LNPs were created, co-expressing the CAR and IL-7—a key hematopoietic cytokine supporting T-lymphocyte homeostasis and survival. Concurrently, a combination with anti-PD-1 antibodies was employed to prevent T-cell exhaustion by blocking the PD-1/PD-L1 inhibitory signaling axis. This combined approach significantly enhanced the proliferation and survival of CAR-T cells, as evidenced by an expansion of the central memory-like T-cell (Tcm) pool. In experimental models, a substantial increase in tumor infiltration by CD8 + T lymphocytes and enhanced functional activity of the infiltrating lymphocytes were observed, ultimately leading to a statistically significant prolongation of survival in mouse melanoma models compared to monotherapeutic approaches [5].

A complementary, extrinsic strategy utilizes administered BNPs to modulate the activity of already infused CAR-T cells. It is possible to develop BNPs that carry activator molecules on their surface or, conversely, inhibitory ligands. Injection of such nanoparticles will allow for temporarily enhancing or suppressing the activity of CAR-T cells on demand, providing control over side effects [62,63]. A prominent example of this approach is the development of T-cell membrane-coated nanoparticles (TCMPs) that combine biomimetic properties with bioorthogonal chemistry [13,92] These TCMPs preserve the complete surface protein repertoire of source T cells, including T-cell receptors (TCRs), co-stimulatory molecules, and adhesion proteins such as LFA-1. The biomimetic design enables the nanoparticles to maintain authentic immune recognition capabilities while providing a versatile PLGA-based synthetic core for therapeutic cargo encapsulation. TCMPs exhibit remarkable biomimetic characteristics, including specific binding to antigen-presenting cells and preferential accumulation in lymphoid tissues and tumor microenvironments. The inherent homing capabilities of T-cell membranes enable preferential accumulation in tumor microenvironments, potentially guiding CAR-T cells to tumor sites through chemotactic signals. The bioorthogonal conjugation system allows for precise attachment of targeting ligands and activation molecules without interfering with natural immune recognition processes. Through this bioorthogonal surface engineering, TCMPs can be functionalized with antigen arrays that provide calibrated activation signals to CAR-T cells, preventing excessive cytokine release and reducing the risk of cytokine release syndrome. Experimental evidence demonstrates that TCMPs exhibit superior circulation time and tumor accumulation compared to conventional nanoparticles. The biomimetic properties enable evasion of mononuclear phagocyte system clearance while maintaining active targeting capabilities [97].

**Figure 7:**
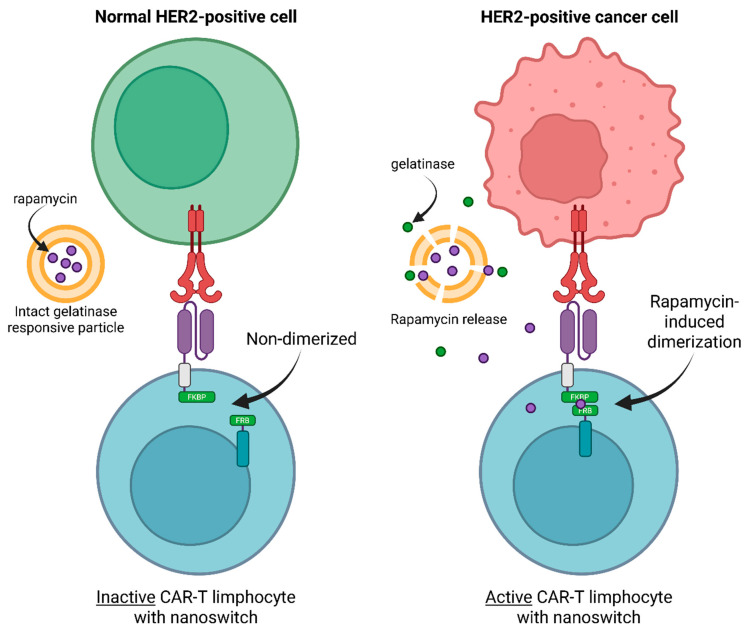
Scheme of the NanoSwitch technology mechanism. Upon entering tumor microenvironment, gelatinase-sensitive nanoparticles encounter gelatinase and release rapamycin. Rapamycin is taken up by CAR-T cells and facilitates activation of CAR-T cells in the tumor but not in healthy tissues.

To address the issue of uncontrolled and non-targeted activation of CAR-T cells, a remote-control platform for their activity was developed, based on the use of a molecular switch called NanoSwitch technology. NanoSwitch (Figure 7) utilizes a modified CAR with FKBP-FRB rapamycin inducible dimerization system in between CD3z and costimulatory domain. The system defaults to inactive state due to absence of rapamycin. However, alongside CAR-T the patient also receives a dose of gelatinase-sensitive nanoparticles that contain rapamycin. Gelatinases are rarely expressed in normal tissues but prevalent in some types of cancers. Gelatinase-sensitive particles are destroyed by tumor gelatinases, causing the release of rapamycin and subsequent activation of CAR-T cells. This allows to lower the toxicity associated with off-target CAR-T activation since rapamycin is predominantly released into tumor microenvironment. Also, rapid cytokine release is avoided due to gradual activation of CAR-T population [98].

**Figure 8:**
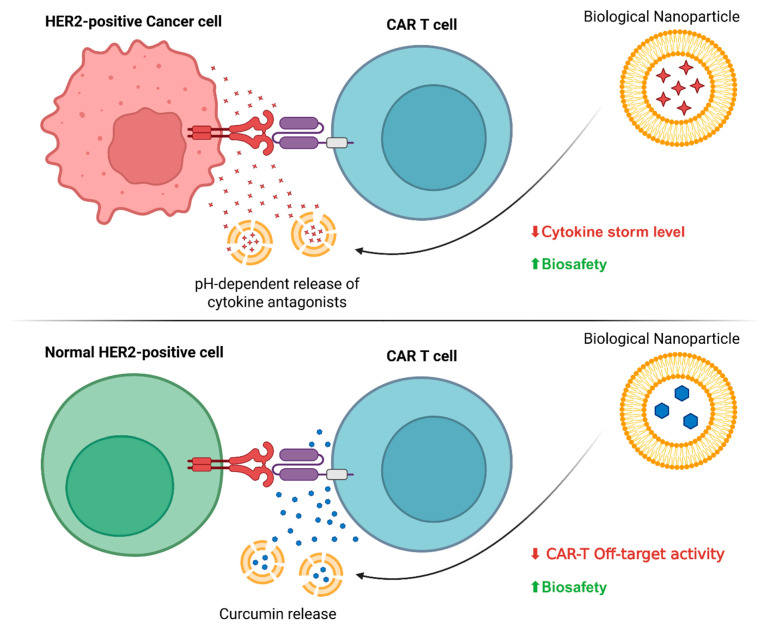
Two complementary nano-modulation strategies for controlling CAR-T cell hyperactivity. The schematic illustrates complementary approaches for managing therapy-induced toxicities. **Upper panel**: Local suppression of cytokine release syndrome (CRS). pH-sensitive nanocarriers accumulate in inflamed tumor microenvironment and release antagonists of pro-inflammatory cytokines (e.g., anti-IL-6), thereby minimizing systemic immunosuppression. **Lower panel**: Direct inhibition of overactivated CAR-T cells. Nanoformulations (e.g., curcumin nanoemulsions) deliver small-molecule inhibitors (NF-κB blockers) intracellularly into T-cells, suppressing their production of TNF-α and other inflammatory mediators.

The main strategies for using nanoparticles to modulate CAR-T cell hyperactivity include two complementary directions: suppression of excessive cytokine response and creation of rapid inhibition systems (Figure 8) [99]. In the first case, special attention is paid to the development of pH-sensitive nanocarriers for the targeted delivery of pro-inflammatory cytokine antagonists [100]. Such systems make it possible to achieve effective suppression of CRS with a significant reduction in systemic immunosuppression compared to standard administration regimens [101]. In parallel, nano formulations of small molecular inhibitors of key pro-inflammatory pathways are being developed. Nanoemulsions of curcumin, which is a natural inhibitor of NF-kB, have shown the ability to significantly reduce the production of TNF-αand other pro-inflammatory mediators by CAR-T cells. An important advantage of such systems is their ability to overcome biological barriers and achieve a high intracellular concentration of the active substance, which is especially important for modulating signaling pathways in activated T cells [102].

## 6. Problems and Drawbacks

Despite the significant potential of remote-control platforms for enhancing the safety of CAR-T cell therapy, their development and clinical translation are associated with a number of fundamental challenges. A primary issue is the dependency of activation systems, such as NanoSwitch, on specific tumor microenvironment enzymes (e.g., gelatinases) [98]. The heterogeneous expression of these biomarkers both between different tumor types and within a single neoplasm can lead to incomplete or uneven activation of CAR-T cells, thereby limiting antitumor efficacy. Furthermore, the release kinetics of the inducer (rapamycin) from the nanoparticles may be insufficient to ensure the rapid and potent antitumor response required to control aggressive malignancies [98,102].

Regarding suppression strategies, the use of nanocarriers for the targeted delivery of cytokine antagonists or anti-inflammatory agents (e.g., curcumin) faces the challenge of timely intervention [101,102]. The pathogenesis of CRS is characterized by a rapid, cascading release of cytokines, which necessitates the preventiv correctede or very early administration of therapeutic nanoparticles for effective syndrome management. In practice, this is difficult to achieve without reliable predictive biomarkers. Moreover, despite targeted delivery, systemic immunosuppression remains a risk, particularly when using potent inhibitors of intracellular signaling pathways that may disrupt not only the function of CAR-T cells but also the activity of endogenous immune cells, which are crucial for long-term antitumor immunity [99,102].

Thus, although nanotechnology offers sophisticated solutions for the spatiotemporal control of CAR-T cells, its successful application requires overcoming limitations related to the biological validation of targets, the kinetics of drug release, and the development of optimal administration protocols to balance efficacy and safety.

Compounding these challenges related to the function and control of CAR-T cells is the critical, yet often underexplored, dimension of immunogenicity associated with the biomimetic nanoplatforms themselves. The convergence of BNPs and CAR therapy holds transformative potential for oncology. However, the clinical translation of this combined approach is critically dependent on a thorough and proactive assessment of immunogenicity risks. A particularly nuanced risk stems from the very feature that grants biomimetic nanoparticles (BMNPs) their functionality: the cell membrane-derived shell. While designed for stealth and targeted delivery, this biological coating can paradoxically act as a catalyst for immune recognition and rejection, potentially compromising both safety and efficacy [103].

Recognition of Allogeneic and Xenogeneic Antigens: BMNPs are typically fabricated using membranes from donor cell lines (e.g., human hematopoietic cell lines) or even non-human sources. These membranes present a full repertoire of Major Histocompatibility Complex (MHC) molecules, blood group antigens, and other surface proteins that can be recognized as foreign by the recipient’s immune system, potentially triggering both cellular and humoral immune responses [103]. This is analogous to a mild allogeneic transplant rejection at the nanoscale. For instance, erythrocyte membrane-coated nanoparticles, while leveraging CD47 for stealth, still present other RBC-specific antigens that can induce antibody formation upon repeated administration, leading to accelerated blood clearance (ABC phenomenon) [104]. An immune response directed against the BMNP shell can have several detrimental outcomes. The development of anti-BNP antibodies can opsonize subsequent doses, leading to their rapid clearance by the reticuloendothelial system (RES), thereby diminishing therapeutic payload delivery and promoting hepatosplenic sequestration [105]. This ABC phenomenon poses a significant barrier to multi-dose regimens, which are often necessary for in vivo CAR-T cell generation or for controlling tumor relapse. Furthermore, cross-reactivity of these antibodies with the patient’s own native cells expressing similar surface markers could, in theory, lead to autoimmune complications, such as transient cytopenias [103]. The immunogenic risk of BMNPs is magnified within the unique inflammatory environment of CAR-T cell therapy. Patients often undergo lymphodepleting chemotherapy and are at high risk for CRS. This pro-inflammatory milieu can heighten innate immune surveillance and lower the threshold for activating adaptive immune responses, potentially turning a mildly immunogenic BMNP into a potent trigger for enhanced immunogenicity against both the nanoparticle and the CAR construct itself [99]. This could exacerbate CRS or contribute to the premature exhaustion of the newly generated CAR-T cells.

## 7. Review of Clinical Progress of the First FDA-Approved CCAR-T Therapies

The development of CAR T-cell therapy represents a transformative advancement in oncology. This section reviews the pioneering FDA-approved CAR-T products—tisagenlecleucel (Kymriah), axicabtagene ciloleucel (Yescarta), and brexucabtagene autoleucel (Tecartus)—which share a common CD19 target but exhibit distinct molecular configurations and clinical profiles [106,107].

### 7.1. Tisagenlecleucel (Kymriah): The First Approved CAR-T Therapy

As the first approved CAR-T product, tisagenlecleucel heralded a new era in cancer immunotherapy. This personalized autologous therapy features a CAR construct targeting CD19 antigen with a distinctive 4-1BB (CD137) co-stimulatory domain, intentionally selected to optimize in vivo persistence and functional activity. The manufacturing process utilizes lentiviral vector transduction of patient-derived T-cells obtained via leukapheresis, underscoring its resource-intensive nature [106].

The mechanism involves redirecting T-lymphocytes against CD19-positive cells, where the 4-1BB domain ensures immediate cytolytic activation while promoting long-term CAR-T cell survival. Clinical validation came from pivotal trials: the ELIANA study in r/r B-ALL demonstrated 81% response rate with majority achieving complete remission, while the JULIET trial in r/r DLBCL showed a 52% overall response rate (ORR) and 40% complete remission (CR) rate [106].

The therapy’s advantages include exceptional efficacy in refractory disease and establishment of long-term immunological control. However, limitations encompass unique toxicities (CRS/ICANS), complex manufacturing logistics, and risk of antigen-negative relapse [106].

### 7.2. Axicabtagene Ciloleucel (Yescarta): Advancing Lymphoma Treatment

Building upon the CAR-T platform, axicabtagene ciloleucel emerged as another CD19-targeted therapy with distinct characteristics. Its molecular architecture incorporates a CD28 co-stimulatory domain rather than 4-1BB, endowing modified cells with potent, rapid proliferation capacity following target engagement. The manufacturing employs gamma-retroviral vector transduction in a multifaceted process involving T-cell activation and expansion [108].

The mechanism emphasizes robust effector cell development capable of eliminating substantial tumor burden rapidly. In the pivotal ZUMA-1 trial focusing on r/r DLBCL, PMBCL, and TFL patients, unprecedented outcomes included 82% ORR and 54% CR, with 42% maintaining remission at 15 months and median overall survival of 25.8 months [108].

Therapeutic advantages include high efficacy in chemorefractory disease and potent cytolytic effect against bulky tumors. Limitations mirror other CAR-T therapies with high toxicity incidence (93% CRS, 64% neurological events), manufacturing complexities, and antigen escape concerns [108].

### 7.3. Brexucabtagene Autoleucel (Tecartus): Optimized for B-ALL

Brexucabtagene autoleucel represents further refinement in CAR-T technology, particularly for aggressive B-cell malignancies. While sharing the CD19 target and CD28 co-stimulatory domain with axicabtagene ciloleucel, its distinguishing feature is an optimized manufacturing protocol incorporating lymphocyte enrichment and selection, yielding a more standardized cellular product using gamma-retroviral transduction [107].

The therapeutic effect manifests through intensive CAR-T cell proliferation and expansion in vivo following CD19 engagement. The ZUMA-3 trial in adult r/r B-ALL demonstrated 71% ORR and 56% CR, with 56% of complete responders achieving MRD-negative status—a significant milestone in leukemia treatment [107].

Advantages include high efficacy in multidrug-resistant B-ALL with deep MRD-negative remissions and the optimized manufacturing process. However, limitations persist with high adverse event incidence (89% CRS, 60% neurological toxicities), complex toxicity management, limited efficacy in extramedullary disease, and antigen-negative relapse risk [107].

### 7.4. Comparative Clinical Implications

Collectively, these first-generation CAR-T therapies demonstrate remarkable efficacy in patients with limited treatment options, yet their distinct co-stimulatory domains (4-1BB versus CD28) translate to different clinical profiles regarding response kinetics and persistence patterns. While all share challenges including toxicity management, manufacturing complexities, and antigen escape, their successive development reflects ongoing optimization in cellular immunotherapy—from pioneering proof-of-concept to refined manufacturing and expanded indications [106,107,108].

## 8. Conclusions

Contemporary CAR therapy, while demonstrating remarkable success in treating oncological diseases, faces several fundamental limitations, including toxicity, insufficient efficacy against solid tumors, and challenges associated with manufacturing and genetic material delivery. In this context, BNPs represent a versatile and promising platform capable of overcoming many of these barriers. The ability of BNPs to enable targeted delivery of genetic constructs, modulate CAR cell activity, and serve as vehicles for in vivo genetic engineering paves the way for next-generation therapies that are safer, more effective, and more accessible.

However, the successful clinical translation of BNP-based technologies necessitates addressing several critical challenges. Key concerns requiring focused attention include batch-to-batch variability in BNP preparations and their rapid clearance by the immune system. Reproducibility of BNP characteristics, such as size, surface charge, and molecular composition, is a prerequisite for their industrial-scale manufacturing and regulatory approval [79]. Furthermore, upon systemic administration, BNPs can be rapidly eliminated by the immune system, which reduces their therapeutic index and delivery efficiency to target tissues [77].

Future advancements in BNP applications for CAR therapy are contingent upon progress in several key research domains.

A primary challenge lies in the standardization of BNP production and analytics. The transition to clinical use necessitates the development of robust, Good Manufacturing Practice (GMP)-compliant protocols to yield highly purified and homogeneous BNP preparations. This entails not only refining purification methodologies but also implementing rigorous characterization using high-resolution techniques such as nanoparticle tracking analysis (NTA), Western blotting, and mass spectrometry. Beyond physicochemical properties, a critical focus must be placed on functional characterization, including empirical assessment of target cell binding specificity in vitro [79].

Concurrently, strategies for surface engineering to circumvent immune clearance are vital for improving the pharmacokinetic profile of BNPs. The objective is to prolong systemic circulation and enhance tumor-specific accumulation. Promising tactics include surface functionalization with polyethylene glycol (PEG) to impart “stealth” properties or the application of membranes derived from a patient’s own autologous cells to create a biologically inspired camouflage [41,109]. An alternative approach involves the encapsulation of BNPs within synthetic lipid nanoparticles, thereby merging the benefits of both biological and synthetic platforms [89].

Furthermore, the development of smart, controllable delivery systems is essential for enhancing the safety profile of CAR therapies. Next-generation BNPs should be engineered for activation by specific tumor microenvironmental cues, such as elevated protease activity or a depressed pH. The scope of control should extend beyond activation to include the on-demand delivery of inhibitory signals, providing a safety switch for CAR cell activity [98,100]. Additionally, BNP-facilitated delivery of gene-editing tools, including CRISPR/Cas systems, for the in vivo genetic engineering of T cells represents a frontier with significant potential [58,92].

Finally, the inherent versatility of BNPs makes them ideal vehicles for combinatorial strategies aimed at overcoming the immunosuppressive tumor microenvironment. They are particularly suited for the targeted co-delivery of immunomodulatory agents—such as checkpoint inhibitors, cytokines, or RNA—directly to the tumor site to act synergistically with CAR therapy. The localized delivery of small molecules designed to disrupt key immunosuppressive pathways, for example, those involving adenosine or indoleamine 2,3-dioxygenase (IDO), via BNPs could markedly improve therapeutic outcomes in solid tumors [102].

In conclusion, while BNPs present a paradigm-shifting opportunity for CAR therapy, their clinical translation hinges on interdisciplinary efforts that address critical issues of manufacturing standardization, precise targeting, and immunogenicity. The trajectory of future research must be directed toward converting promising preclinical proofs-of-concept into viable clinical interventions by refining nanocarrier design and production. The convergence of cellular therapy, nanotechnology, and genetic engineering provides a robust foundation for a new generation of potent, personalized cancer treatments.

## Figures and Tables

**Table 1:** CAR generation evolution based on intracellular domains.

Generation	Intracellular Domains	The Principle of Operation	Advantages	Disadvantages	Citation
First	CD3ζ	Provides only activation signal 1 (via ITAM motives).	Simplicity of construction.	Insufficient proliferation, rapid cell death in vivo, and low efficiency.	[18,19,20]
Second	CD3ζ + one co-stimulatory domain (CD28, 4-1BB, etc.)	Provides signal 1 (activation) and signal 2 (co-stimulation).	Rapid activation, proliferation, and long-term persistence of cells.	Risk of cytokine release syndrome (CRS) and neurological toxicity (ICANS)	[18,19,20]
Third	CD3ζ + two co-stimulatory domains (e.g., CD28 + 4-1BB)	An amplified and prolonged activation signal.	A potentially more powerful response.	Increased risk of depletion of T cells, lack of clear advantages over the 2nd generation in the clinic.	[19,20]
Fourth	Multidomain constructs + additional gene cassettes (e.g., cytokines, induced promoters).	Targeted delivery of immunomodulators to the tumor microenvironment or logical activation (AND-gate).	High safety (reduction in on-target/off-tumor toxicity), overcoming the immunosuppressive microenvironment.	High complexity of design and production, and potential immunogenicity of the structure.	[19,20]
